# Draft Genome Sequence of *Stenotrophomonas maltophilia* Strain EPM1, Found in Association with a Culture of the Human Parasite *Giardia duodenalis*

**DOI:** 10.1128/genomeA.00182-13

**Published:** 2013-04-18

**Authors:** Davide Sassera, Iacopo Leardini, Laura Villa, Francesco Comandatore, Claudio Carta, André Almeida, Maria do Céu Sousa, Stefano Gaiarsa, Piero Marone, Edoardo Pozio, Simone M. Cacciò

**Affiliations:** DIVET, Università degli Studi di Milano, Milan, Italya;; Department of Infectious, Parasitic, and Immunomediated Diseases, Istituto Superiore di Sanità, Rome, Italyb;; National Center for Rare Diseases, Istituto Superiore di Sanità, Rome, Italyc;; Instituto de Ciências e Tecnologias Agrárias e Agroalimentares da Universidade do Porto, Porto, Portugald;; Faculdade de Farmácia/CEF, Universidade de Coimbra, Coimbra, Portugale;; Fondazione Policlinico IRCCS San Matteo, Pavia, Italyf

## Abstract

We report the draft genome sequence of the *Stenotrophomonas maltophilia* strain EPM1, found in association with a culture of *Giardia duodenalis*. The draft genome sequence of *S. maltophilia* strain EPM1, obtained with Roche 454 GS-FLX Titanium technology, is composed of 19 contigs totaling 4,785,869 bp, with a G+C content of 66.37%.

## GENOME ANNOUNCEMENT

*Stenotrophomonas maltophilia* is an aerobic, nonfermentative gammaproteobacterium commonly found in water and soil and in association with plants. *S. maltophilia* has several beneficial effects on plants, including protection from pathogens, promotion of growth, and biodegradation of pollutants (1). Furthermore, it is recognized as an emerging opportunistic human pathogen that is spread easily in hospital settings (2). In immunocompromised patients, *S. maltophilia* can lead to nosocomial infections with a significant fatality-to-case ratio (3), and up to 15% of patients with cystic fibrosis are colonized with *S. maltophilia* (4). Different *S. maltophilia* strains exhibit resistances to common antibiotics due to chromosomally encoded multidrug resistance proteins, such as antibiotic-inactivating enzymes and efflux pumps (5). These resistances are suggested to be acquired in the environment (6).

*S. maltophilia* strain EPM1 was found during the sequencing of two human-derived strains of *Giardia duodenalis*, a flagellated protozoan that parasitizes the small intestine of mammals. In its vegetative stage, this parasite can be grown in a medium supplemented with antibiotics and antifungals. The presence of *S. maltophilia* is likely the result of laboratory contamination of this culture medium. The environmental origin of the EPM1 strain is reinforced by its occurrence in other *G. duodenalis* strains that were propagated in the same medium. Here, we present the sequence and annotation of the genome of *S. maltophilia* EPM1. Whole DNA was extracted using a commercial kit (Qiagen) and was subjected to quality controls. Next-generation sequencing was performed on a full plate of the Roche 454 GS-FLX Titanium platform (7). A total of 1,290,645 reads were generated, with an average length of 477 bases, for a total of 591 megabases. A first assembly was performed by feeding all the reads to MIRA 3.4 (8). The 1,804 contigs obtained were subjected to BLAST analysis against *Giardia* and *Stenotrophomonas* databases. Reads belonging to contigs exhibiting an E value of <10^-4^ against the *Giardia* database but not against the *Stenotrophomonas* database were excluded from subsequent analyses. The remaining 816,591 reads were assembled on MIRA 3.4. The resulting assembly of 258 contigs (with an average coverage of 70.44-fold in contigs of >5,000 bp) was subjected to an in-house finishing procedure using the Gap4 (9) and NUCmer (10) software, allowing a total of 239 unequivocal contig joins. The resulting assembly consists of 19 contigs with a G+C content of 66.37%, for a total of 4,785,869 bp.

Multilocus sequence typing (MLST) analysis was performed on PubMLST (http://pubmlst.org) (11) and showed that *S. maltophilia* EPM1 differed from codified sequence types, yet it clustered with clinical isolates in genogroup 6 in phylogenetic analyses (12).

Annotation was performed automatically on the RAST server (13) using Glimmer base calling. The genome includes 4,334 predicted coding sequences and 75 RNAs. Reading frames obtained from the RAST annotation were subjected to BLAST analysis against the Comprehensive Antibiotic Resistance Database (CARD; Michael G. DeGroote, Institute for Infectious Disease Research, McMaster University, Hamilton, Ontario, Canada [http://arpcard.mcmaster.ca]). This approach highlighted the presence of 154 genes related to antibiotic resistance.

### Nucleotide sequence accession numbers. 

This Whole Genome Shotgun project has been deposited at DDBJ/EMBL/GenBank under the accession no. AMXM00000000. The version described in this paper is the first version, accession no. AMXM01000000.
